# Predictors of work engagement among rural and remote nurses who provide hospice, palliative, and end of life care: Results from a national study

**DOI:** 10.1371/journal.pone.0358592

**Published:** 2026-09-16

**Authors:** Kelly L. Penz, Erin Barker, Julie G. Kosteniuk, Norma J. Stewart, Steinunn Jónatansdóttir, Martha L. P. MacLeod

**Affiliations:** 1 College of Nursing, University of Saskatchewan, Saskatoon, Saskatchewan, Canada; 2 School of Nursing, University of Northern British Columbia, Prince George, British Columbia, Canada; 3 Canadian Centre for Rural and Agricultural Health, University of Saskatchewan, Saskatoon, Saskatchewan, Canada; 4 University of Akureyri, Norðurslóð, Akureyri, Iceland; King Khalid University, SAUDI ARABIA

## Abstract

**Background:**

The availability of palliative health care professionals is considered a global concern, especially within rural/remote practice settings. Nurses across all domains of practice are expected to advocate for high-quality hospice, palliative, and end of life (H/P/EOL) care. However, within many rural/remote geographical areas, formal palliative care services are non-existent, leaving generalist nurses to take on this complex responsibility.

**Methods:**

Examining results from a Pan-Canadian cross-sectional survey of rural and remote nurses (N = 3,822), this paper explores a subset (n = 295) of nurses who provided hospice, palliative and/or end of life care as part of their practice. Analyses examined job demands and job resources, predictors of work engagement, and a summary of open-ended responses.

**Results:**

The rural and remote nurses who worked in hospice, palliative, and/or end of life care had significantly higher satisfaction with practice demands related to their safety, lower practice resources related to staffing and time, and higher levels of work engagement compared to nurses in other areas. Multiple regression analyses demonstrated that seven variables accounted for 40% of the variance in their work engagement, including: perceived mental health, job satisfaction, interprofessional collaboration, affective organizational commitment, continuance organizational commitment, normative organizational commitment, and lower job demands related to working conditions. Open-ended data revealed that rural nurses felt privileged to provide H/P/EOL care, however, faced barriers related to blurred personal/professional boundaries when dealing with death/dying and lack of access to palliative resources.

**Conclusions:**

This is the first Canadian national profile of rural and remote nurses who provide hospice, palliative and end of life care and highlights key areas related to their professional quality of life (e.g., work engagement, organizational commitment, job resources/demands). The results of this analysis may inform practice and policy development for health human resource planning and recruitment/retention in hospice, palliative and/or end of life care across rural and remote settings.

## Introduction

Work engagement is characterized by a positive sense of vigor, dedication, and absorption in one’s work [1,2], and is an important determinant of occupational well-being, particularly in demanding practice settings such as hospice, palliative and end of life (H/P/EOL) care nursing. Nurses across all domains of practice are expected to advocate for high-quality H/P/EOL care, however, the availability and sustainability of hospice and palliative health care professionals is considered a global concern [3]. Furthermore, in rural and remote geographical areas, formal palliative care services are often limited or non-existent, leaving generalist nurses with less experience or education in hospice and palliative nursing to take on this complex responsibility.

Rural and remote nurses provide H/P/EOL care as part of a broader generalist-specialist scope of practice and face unique challenges such as less frequent encounters involving death/dying, more isolated work settings, resource limitations, and shortages of practitioners with expertise in this critical area of practice [4,5]. Even with these challenges, these nurses are expected to provide competent compassionate care to individuals with life-limiting illnesses, including interacting and building trust with family members who are facing difficult decisions in high stress situations. Rural and remote nurses involved in H/P/EOL practice need to feel supported in order to maintain high levels of work engagement and confidence, which may have a direct impact on the quality of care they are able to deliver. Developing a better understanding of the potential predictors of work engagement among rural and remote nurses who provide H/P/EOL care on a national level is essential.

## Background

The proportion of Canadian nurses who work in rural and remote settings has continually declined over the past decade (from 11.1% in 2013 to 9.6% in 2022) and has not kept pace with the growth of the aging population in these settings [6]. Population aging will continue to accelerate over the next two decades, with those aged 85 and over increasing rapidly (e.g., baby-boomer cohort) and the majority facing both chronic and acute illnesses, requiring increased access to health care services [7]. Conditions such as dementia, cardiovascular disease, and cancer are also becoming more prevalent among older adults [8], all of which benefit from a H/P/EOL approach to care. It is evident that provision of care for those with life-limiting illness or terminal diagnoses will become more commonplace for nurses who practice in rural and remote geographical settings.

### Hospice, palliative, and end of life care

Hospice palliative care is a unique and complex area of health care that focuses on the relief of suffering (e.g., pain, anxiety, dyspnea) and improved quality of life for those facing life-limiting illness and their family caregivers [9–11]. Palliative care is considered an umbrella term that places the person receiving care and their family members at the centre of decision making throughout the illness trajectory. Hospice and end of life care fit under the palliative approach to care, but typically focus on the physical and psychosocial care that are provided in the final months or weeks of life and into the bereavement period. Although it is well recognized that early access to palliative care improves quality of life, referrals to palliative care often occur late in the illness trajectory; reinforcing fears and misconceptions that palliation is only associated with hopelessness, withdrawal of treatment, and death/dying [12]. There is a similar lack of understanding of the benefits of palliative care among health care professionals, with models of palliative care increasingly focused on improving palliative care competencies among non-specialist health care providers and increasing remote consultation capacity for rural and remote geographical settings [10,13,14]. Globally, palliative care is recognized as a human right to health, however, insufficient access to specialty palliative and end of life care services remains an ongoing problem [11], especially in rural and remote settings [15].

### Realities of hospice palliative care nursing in rural and remote settings

In a systematic review of 14 studies involving 1,820 patients, key elements identified that improved palliative care outcomes in rural settings included continuity and coordination; prepared, informed, and motivated health care teams; and access to clinicians with palliative care expertise [16]. A recent scoping review of 53 studies support these findings and identified additional facilitators to accessing palliative care in rural and remote settings including advanced care planning, improved professional collaboration (e.g., between nurses and primary care practitioners), specialized education and training, and utilization of virtual care tools (e.g., telehealth) [17]. Although rural and remote nursing is consistently recognized as a generalist-specialist area of practice [18,19], few have access to formal continuing educational opportunities in H/P/EOL care and/or the ability to collaborate with multidisciplinary team members with expertise in this area [4,5]. In Canada, decolonization of palliative care (e.g., challenging inequities) and addressing systemic racism and different cultural beliefs surrounding death and dying are also key aspects of nursing care within rural and remote communities [20]. However, building partnerships with Indigenous communities and embracing cultural safety in the provision of palliative care are complex and continues to be a challenge for rural nurses who practice in these settings [21].

Research suggests that nurses who provide H/P/EOL care may experience higher levels of stress, especially in settings where they are more isolated, are required to continually adapt to new situations, and face challenges with accessing supports in a timely manner [22]. There is need for additional structural [13] and technological [23] resources for rural H/P/EOL settings. Persistent barriers to providing high quality H/P/EOL care highlight the need to recognize the physical, mental, and emotional toll that rural and remote nurses face while working in the presence of suffering/grief and loss and how they can best be supported to remain engaged in their practice.

### Work engagement and the job demands-resources model

Work engagement is a well-recognized, multidimensional construct in occupational health research focused on one’s psychological connection to their work through a positive sense of vigor, dedication, and absorption [1,2]. Research exploring work engagement among various occupational groups has highlighted the importance of the Job Demands-Resources (JD-R) Model [24,25], which integrates elements of potential work-related stressors and motivators. Within this model, job demands are defined as the “physical, social, or organizational aspects of the job that require sustained physical and/or psychological effort or skills and are associated with certain physiological and/or psychological costs (p. 312)” [26]. Job resources are recognized as having the ability to buffer or reduce the impact of job-related demands [24] and are defined as the “physical, psychological, social, and organizational aspects of the job that are either functional in achieving work goals…and the associated physiological and psychological costs, or stimulate personal growth, learning, and development (p.312)” [26]. Job demands and resources in nursing practice are typically viewed as encompassing various combinations of adaptable antecedents of work engagement [27] at the organizational (e.g., working conditions, professional development) [28–30], interpersonal/ interprofessional (e.g., collegial support) [29–31], and position/task level (e.g., autonomy, performance feedback) [29,30,32,33].

### Work engagement in professional nursing practice

A growing body of nursing literature has recognized the importance of work engagement as it correlates to job resources and demands, burnout, turnover, organizational commitment, retention, perceived competence, and improved patient outcomes [29,34–39]. A systematic review of 18 studies on work engagement in professional nursing practice identified over 70 influencing factors on nurses’ work engagement categorized into six themes: 1) organizational climate, 2) job resources, 3) professional resources, 4) personal resources, 5) job demands, and 6) demographic variables [29].

Despite the mounting evidence on the importance of work engagement among nurses and other health care professionals, the majority of samples are drawn from nurses who work in urban settings or do not differentiate between rural and urban nurse participants. Furthermore, few studies have specifically focused on rural and remote nurses who provide H/P/EOL care, especially from a national perspective. Identifying the factors that contribute to work engagement in this context is crucial for developing rural specific interventions that can enhance nurses’ job satisfaction, reduce burnout, and improve retention among rural and remote nurses who provide H/P/EOL care.

The aims of this paper are to develop a better understanding of a national subset of rural and remote nurses who provide H/P/EOL care and to examine potential predictors of their overall work engagement. Our goal through this work is to share knowledge that has the potential to inform strategies and policies that support nurses in accessing palliative educational opportunities. We also hope to inform policies that increase understanding and engagement of rural and remote nurses in this important area of practice, advocate for earlier access for patients who could benefit most, and ultimately promote better patient care and outcomes.

## Methods

### Research design

Examining data from a pan-Canadian cross-sectional survey of regulated nurses in rural and remote practice settings (N = 3,822) [40], this paper explores and compares a subset (n = 295) of nurses who provided hospice, palliative and/or end of life care as part of their practice. For the full details and information about the sampling frame, survey questionnaire development, data collection period (April 2014 to August 2015), and methodology of the original study, see [40].

This study was conducted in accordance with the Canadian Tri-Council Policy Statement: Ethical Conduct for Research Involving Humans (TCPS 2) [41]. Ethical approval for the study was received from the Behavioural Research Ethics Boards (REB) of the University of Northern British Columbia [E2013.0320.037.00], University of Saskatchewan [13–222], University of Lethbridge [2013–047], Laurentian University, Hôpital Maisonneuve Rosemont (affiliate of the Université de Montréal) [13046], Dalhousie University [2013–3131], Aurora College [15426], Nunavut Research Institute [05 007 14N-M], and the Prince Edward Island Research Ethics Board. Each study participant provided voluntary informed consent. Specifically, a consent statement was included at the start of the survey outlining information about the study: participants’ rights to voluntary participation, freedom to withdraw, confidentiality, and anonymity. Participants who returned a completed questionnaire indicated their consent to participate in the national study, with the choice to not complete or return the survey considered withdrawal from the study. The STROBE checklist for cross-sectional studies guided the reporting of this analysis [42].

### Study setting and sampling

The original study used a multi-level systematic, stratified sampling frame including: (1) random sampling of Registered Nurses (RNs), Nurse Practitioners (NPs), Licensed or Registered Practical Nurses (LPNs), and Registered Psychiatric Nurses (RPNs) with rural postal code sets within each of the 10 Canadian provinces; (2) all rural and remote NPs; and (3) all nurses RNs, NPs, LPNs, and RPNs working in the territories (Yukon Territory, Northwest Territory, and Nunavut) [39]. This resulted in a stratified random sample of 3,822/9,622 (40% response rate) eligible regulated rural and remote nurses including RNs, NPs, LPNs, and RPNs. The Statistics Canada definition of Rural and Small Town Canada [43] was used to define both rural and remote and Northern communities. Thus, “rural” referred to communities with a population of less than 10,000 people, and included those communities where less than 50% of the employed population commute to larger urban communities for work [40]. The sampling frame was developed to achieve statistically significant results (95% confidence interval) with a margin of error of 0.05 for the 10 provinces and three territories across Canada. Due to the generalist (i.e., broad range, multi-specialist across the lifespan) nature of practice for rural and remote nurses, respondents were asked a survey question: What is your area of current practice?, with “mark all that apply” to the following areas: 1) Acute care, primary care, 2) community health, 3) long-term care, 4) home care, 5) hospice/end of life/palliative care, 6) mental health, and 7) other. The subsample of n = 295 for this analysis are those respondents who indicated that hospice/end of life/palliative care is an area of current practice. It is important to note that it was not possible to more accurately define this subsample beyond use of the single, self-reported area of current practice with the option to “mark all that apply.” It is possible that other respondents had provided H/P/EOL care in the past, but did not consider it a current area of practice and were not included in the analysis.

### Data collection

The original survey questionnaire (27-page paper and online formats; [40]) was developed and pilot-tested by a 16-member research team and 19-member advisory group of nurse leaders in rural and remote practice. The survey questionnaire consisted of five main sections of individual characteristics, work community, workplace, nursing practice, and personal/professional well‐being. Data were collected between June 2014 to August 2015, with all provincial and territorial nursing regulatory bodies assisting in mailing and online distribution of the research package and questionnaire following Dillman’s Tailored Design Method and repeated follow-up [44]. The paper and online questionnaires were available in both English and French languages.

### Instruments/Measures, variables, and open-ended data included in the analyses

A number of standardized scales, newly developed scales, and single-item variables were incorporated into the survey questionnaire and included in the statistical analyses. In addition, open-ended data arising from two questions included at the end of the survey questionnaire were examined: 1) What does it mean to be a nurse in rural and/or remote Canada?, and 2) Please share any other comments about your experience of nursing in rural and/or remote communities. Demographic, workplace/nursing practice, and work community variables were used to describe and compare those rural and remote nurses who provide H/P/EOL care and the remaining respondents who do not consider this a part of their practice.

For the predictors of work engagement regression analyses, the dependent variable of work engagement was measured with the Utrecht Work Engagement (UWE) Scale- Short Form [2] worded in the survey as: Read each statement and decide if you ever feel this way about your work. The 9 items related to work engagement (e.g., I am enthusiastic about my job, I am proud of the work that I do) were scored from never to everyday (scores ranging from 0–54), with a Cronbach’s alpha 0.91 for this sample indicating high internal consistency. In addition, a total of 25 potential predictor variables of work engagement were considered for this analysis based on the sample size of n = 295 nurses who provide H/P/EOL care, relevant literature, and insights from the research team. The number of potential predictors was based on guidelines recommending between 10–15 participants per predictor variable for model selection techniques in multiple regression analyses [45]. The Collaborative Pan-Canadian Health Human Resource Planning Framework [46] was used to organize the 25 potential independent variables at the individual, workplace/nursing practice, and work community level as predictors of work engagement among rural and remote nurses who provide H/P/EOL care. Table 1 outlines the variables considered for inclusion in the regression analysis including scale/item information and scoring, reliability estimates (where applicable), and source. For all scales included, higher scores indicated a higher level of the construct or attribute.

**Table 1:** Potential predictors of work engagement, scales/items, scoring, source, and reliability estimates.

Measure	Items/ Scoring/ Reliability Estimates	Source
**Individual: Demographics and Health**		
Gender (A1)	What is your gender? (Female, Male)	Developed for this study
Age (A2)	What is your year of birth? (year of survey completion minus recorded year of birth)	Developed for this study
Mental Health (L2)	In general, I would say my mental health is: (1 item, 5 point Likert scale from poor, fair, good, very good, excellent)	Developed for the study
Perceived Stress (l8SUM)	How often you felt or thought a certain way (4 items, 5-point Likert scale from never to very often, range of scores 4–20) H/P/EOL sample Cronbach’s α = 0.75, Cronbach’s alpha coefficients range from 0.60 to 0.82 across studies	Perceived Stress Scale [47]
Burnout L4	I feel burned out from my work (1 item, 7-point Likert scale from never to always)	[48]
**Workplace/ Nursing Practice**		
Satisfaction with current nursing practice (E1)	Overall, I am satisfied with my current nursing practice (1 item, 5-point Likert scale from strongly agree to strongly disagree)	Developed for this study
Perceived confidence in work (E14)	I would describe my level of confidence in my work as: (1 item, 4-point Likert scale from extremely high, somewhat high, somewhat low, extremely low)	Developed for this study
Interprofessional Collaboration (H2)	Thinking about interprofessional collaboration in your primary nursing position, indicate the choice that best describes your feelings about the statement: I am able to share and exchange ideas in a team discussion (1 item, 7 point Likert scale from not at all, to a small extent, to a moderate extent, to a fairly great extent, to a great extent, to a very great extent, and not applicable)	Developed for this study
Organizational commitment subscale: Affective commitment to the organization (ACOsum)	With respect to your own feelings about your primary workplace (4 items, 7-point Likert scale from strongly disagree to strongly agree) H/P/EOL sample Cronbach’s α = 0.84, Cronbach’s alpha coefficients with original 6 item subscale range from 0.80 to 0.90 across studies	Modified (reduced from 18 to 12 items, 4 items per subscale) from [49]
Organizational commitment subscale: Continuance commitment to the organization (CCOsum)	Same as above (4 items) H/P/EOL sample Cronbach’s α = 0.65, Cronbach’s alpha coefficients with original 6 item subscale range from 0.60 to 0.80 across studies	Modified (reduced from 18 to 12 items, 4 items per subscale) from [49]
Organizational commitment subscale- Normative commitment to the organization (NCOsum)	Same as above (4 items) H/P/EOL sample Cronbach’s α = 0.71, Cronbach’s alpha coefficients with original 6 item subscale range from 0.70 to 0.80 across studies	Modified (reduced from 18 to 12 items, 4 items per subscale) from [49]
JRIN subscale: Supervision, recognition, and feedback (I1asum)	Thinking of your primary workplace and primary work community, please indicate your level of agreement with the following (4 items, 5-point Likert scale from strongly agree to strongly disagree, and not applicable) H/P/EOL sample Cronbach’s α = 0.88	Job Resources in Nursing (JRIN) scale developed for this study [30]
JRIN subscale: Collegial support (I1bsum)	Same as above (4 items) H/P/EOL sample Cronbach’s α = 0.78	Developed for this study [30]
JRIN subscale: Staffing and Time (I1csum)	Same as above (4 items) H/P/EOL sample Cronbach’s α = 0.78	Developed for this study [30]
JRIN subscale: Training, professional development, and continuing education (I1esum)	Same as above (4 items) H/P/EOL sample Cronbach’s α = 0.87	Developed for this study [30]
JRIN subscale: Autonomy and control (I1fsum)	Same as above (4 items) H/P/EOL sample Cronbach’s α = 0.76	Developed for this study [30]
JDIN subscale: Preparedness for scope of practice (I1hsum)	Same as above (4 items) H/P/EOL sample Cronbach’s α = 0.76	Job Demands in Nursing (JDIN) scale developed for this study [30]
JDIN subscale: Isolation (I1jsum)	Same as above (3 items) H/P/EOL sample Cronbach’s α = 0.41	Developed for this study [30]
JDIN subscale: Comfort with working conditions (I1ksum)	Same as above (4 items) H/P/EOL sample Cronbach’s α = 0.68	Developed for this study [30]
JDIN subscale: Safety (I1lsum)	Same as above (4 items) H/P/EOL sample Cronbach’s α = 0.68	Developed for this study [30]
**Community**		
Population of primary work community (C4)	What is the population of your primary work community? (1 item, 8 categories 499 or less, 500–999, 1,000–2,499, 2,500–4,999, 5,000–9,999, 10,000–29,999, 30,000–99,999, 100,000 or over)	Developed for the study
Distance to basic referral centre (c12A)	How far is your primary work community from the closest basic referral centre (e.g., community with basic specialty services such as general internal medicine, general surgery, ophthalmology, orthopedic surgery, radiology) (Kilometers 0–99, 100–199, 200–499, 500–999, 1000 or more)	Developed for the study
Work community cohesion (c13sum)	Thinking about your primary work community, please indicate your level of agreement with the following statements (9-items, 5-point Likert scale from Strongly disagree to strongly agree) H/P/EOL sample Cronbach’s α = 0.92	Developed for this study
Personal/professional boundaries between the workplace and community (C14sum)	Thinking about your primary workplace and primary work community, please indicate your level of agreement with the following statements (3 items, 5-point Likert scale from strongly agree to strongly disagree) H/P/EOL sample Cronbach’s α = 0.67	Developed for this study
Satisfaction with work community (C16)	Overall, I am satisfied with my primary work community (1 item, 5-point Likert scale from strongly agree to strongly disagree)	Developed for this study

### Data analyses

Prior to descriptive and inferential analyses, case means were imputed for missing values for each subscale of the JRIN and JDIN for participant’s subscales that were missing 25% or less (i.e., one item), and discarded if missing greater than 25% of items [29]. Descriptive (e.g., frequencies, percentages) and inferential (Chi-square, t tests) statistics were used to describe and compare the sample of rural and remote nurses who provide H/P/EOL care and the remaining sample who did not indicate that they provide this care as part of their practice, with a set alpha of 0.05 for interpretation. Prior to running the multiple regression, the relationship between each of the 25 potential independent variables and the outcome variable of work engagement were analyzed using Pearson *r* correlations for continuous data, Chi-square for categorical data, and examination of the internal consistency reliability of applicable scales. To reduce the chance of making a Type I error, an alpha of *p* < 0.01 was used for determination of inclusion of variables in the regression analysis. As recommended, all correlations between the dependent variable and independent variables were higher than the correlations observed between each set of independent variables [50]. The data for the analysis was normally distributed and met the assumptions of multiple regression including: linearity, homoscedasticity, singularity, and the absence of multicollinearity. To describe and illustrate features of nursing work in a rural setting, open-ended data from the H/P/EOL care sample were analyzed descriptively [51] to focus on experiences of rural nurses caring for this population.

## Results

### Sample characteristics

Out of the full national sample of rural and remote nurses, only a small proportion (295/3822, approximately 8%) indicated that H/P/EOL care was part of their practice The rural and remote nurses who considered H/P/EOL as an area of practice were significantly younger than the Non-H/P/EOL sample, with an almost half-and-half split of the H/P/EOL care sample being RNs (48%) or LPNs/Registered Practical Nurses (50%) (Table 2). The majority of both the H/P/EOL and Non-H/P/EOL rural and remote nurses were female, with no significant differences in the proportions of male and female nurses between groups. The H/P/EOL care nurses were significantly more likely to be working in community-based settings, in the smallest communities (population of 999 or less); with lower proportions working in hospital settings compared to the Non-H/P/EOL rural and remote nurses. The rural and remote nurses who worked in H/P/EOL care were also significantly more likely to have experienced violence and/or witnessed violence (both physical assault and emotional abuse) in the last 4 weeks compared to Non-H/P/EOL care nurses.

**Table 2:** Sociodemographic and workplace characteristics of rural and remote nurses who provide H/P/EOL care and rural and remote nurses who do not provide H/P/EOL care.

	H/P/EOL n = 295 *n* (%)	Non-H/P/EOL n = 3527 *n* (%)	Chi-square *P*-value
**Gender**			
Male	23 (8.1)	211 (6.2)	0.210
Female	260 (91.9)	3175 (93.8)	
**Age (years)**			**0.002**
< 25	11 (3.9)	64 (1.9)	
25-34	66 (23.2)	554 (16.7)	
35-44	56 (19.7)	656 (19.7)	
45-54	72 (25.4)	975 (29.3)	
55-64	75 (26.4)	926 (27.9)	
> 64	—	148 (4.5)	
**Nurse Type**			**<0.001**
RN	141 (47.8)	1923 (55.1)	
LPN/Reg Practical nurse	146 (49.5)	1209 (34.7)	
Nurse Practitioner/Reg Psychiatric nurse	8 (2.7)	357 (10.2)	
**Region of primary employment**			**<0.001**
British Columbia/Alberta	41 (13.9)	609 (17.5)	
Saskatchewan/Manitoba	59 (12.0)	776 (22.2)	
Ontario	21 (7.1)	397 (11.4)	
Quebec	62 (21.0)	249 (7.1)	
Maritimes	71 (24.1)	884 (25.3)	
Yukon/Northwest Territories/Nunavut	41 (13.9)	574 (16.5)	
**Place of employment**			**0.001**
Community-based health care	173 (58.8)	1661 (48.1)	
Hospital	47 (16.0)	885 (25.6)	
Long-term care/Nursing home	59 (20.1)	715 (20.7)	
Other (e.g., primary care)	15 (5.1)	195 (5.6)	
**Length of time in primary position**			**0.030**
2 years or less	64 (21.7)	814 (23.3)	
3-5 years	68 (23.1)	732 (21.0)	
6-9 years	59 (20.0)	578 (16.6)	
10-14 years	40 (13.6)	418 (12.0)	
15-19 years	8 (2.7)	230 (6.6)	
20 + years	6 (2.0)	684 (19.6)	
**Population of work community**			**0.001**
999 or less	54 (18.9)	445 (13.2)	
1,000-9,999	167 (58.6)	1845 (54.7)	
Over 10,000	64 (22.5)	1082 (32.1)	
**Do you pronounce death**			**<0.001**
Yes	152 (51.5)	1160 (33.2)	
No	143 (48.5)	2329 (66.8)	
**Intent to leave position in next 12 months**			0.394
Yes	84 (29.1)	898 (26.8)	
No	205 (70.9)	2459 (73.2)	
**Experienced violence- (physical assault in the last 4 weeks)**			**<0.001**
Yes	92 (31.7)	733 (21.9)	
No	198 (68.3)	2620 (78.1)	
**Experienced violence- (emotional abuse in the past 4 weeks)**			**0.012**
Yes	121 (41.7)	1152 (34.4)	
No	169 (58.3)	2201 (65.6)	
**Witnessed violence (physical assault in the past 4 weeks)**			**0.005**
Yes	101 (35.1)	913 (27.3)	
No	187 (64.9)	2426 (72.7)	
**Witnessed violence (emotional abuse) in the last 4 weeks)**			**0.039**
Yes	123 (42.7)	1222 (36.6)	
No	165 (57.3)	2117 (63.4)	

Blank cells indicate cell size ≤ 5. Bold font indicates significance level of p ≤ 0.05

Not all categories add up to total sample size due to missing data.

When comparing mean scores across the job resources and job demands subscales, the H/P/EOL rural and remote nurses had significantly lower job resources related to staffing and time, and significantly higher job demands related to their safety when compared with rural and remote nurses in other areas of practice (Table 3). Even in the face of these challenges, they experienced significantly higher levels of work engagement when compared to rural and remote nurses in other areas of practice.

**Table 3:** Comparison of job resources in nursing, job demands in nursing, and work engagement for rural and remote nurses who provide H/P/EOL care and rural and remote nurses who do not provide H/P/EOL care.

	H/P/EOL n = 295	Non-H/P/EOL n = 3527	
T-Test^†^	Means (SD)		*P*-value
JRIN Summated	77.92 (13.55)	79.89 (12.88)	**0.021**
JRIN Subscales			
Supervision, Recognition, and feedback	12.87 (3.88)	12.93 (3.88)	0.822
Collegial support	16.15 (2.62)	15.94 (2.59)	0.197
Staffing and time	10.92 (3.72)	11.65 (3.57)	**<0.001**
Technology	12.90 (3.39)	13.29 (3.24)	0.064
Training, PD, continuing education	12.32 (3.65)	12.71 (3.43)	0.066
Autonomy and control	13.17 (3.17)	13.53 (3.04)	0.056
JDIN Summated	51.25 (9.90)	50.93 (9.94)	0.652
JDIN Subscales			
Work-related travel	9.65 (3.84)	9.70 (3.61)	0.855
Preparedness/Scope of practice	7.66 (1.92)	7.80 (2.02)	0.245
Equipment and supplies	7.49 (2.62)	7.33 (2.50)	0.290
Isolation	6.90 (1.94)	6.76 (2.00)	0.278
Comfort with working conditions	9.29 (2.53)	9.30 (2.38)	0.927
Safety	10.43 (3.07)	9.92 (2.96)	**0.005**
Work Engagement	40.30 (8.99)	38.40 (9.41)	**<0.001**

Bold font indicates significance level of *p* ≤ 0.05

† Independent samples t-test, two-tailed significance with equal variances assumed.

Terms: Job Resources in Nursing (JRIN), Job Demands in Nursing (JDIN), Professional Development (PD)

### Predictors of work engagement among rural nurses who provide H/P/EOL

A total of 7/25 independent variables were excluded from the multiple regression analyses due to insignificant relationships/correlations with work engagement, including: Individual (age, gender), Workplace/ Nursing Practice (perceived confidence; job resources related to training, professional development, and continuing education), and Community (population of primary work community, distance to basic referral centre, and personal/professional boundaries between the workplace and community). Three additional independent variables were excluded due to low internal consistency reliability or presence of multicollinearity (i.e., Job resources related to collegial support, job demands related to isolation, and job demands related to safety). The remaining 15 potential predictors were significantly correlated with the outcome variable of work engagement, with correlations ranging from +/- 0.199–0.439.

Table 4 outlines the results of the multiple regression analysis. Of the 15 predictors included in the model, 8 were theoretically relevant based on prior literature and bivariate analyses, however, were not statistically significant (p > 0.05). This suggests that their independent effects were not detectable in this sample after controlling for the remaining predictors. The *p*-values for these 8 variables were included in Table 4 for transparency and to assist with evaluation of potential cofounders in the model. The remaining 7 out of the 15 variables were significant predictors (*p ≤* 0.05), accounting for 40% of the total variance (adjusted *R*^2^ = 0.398) in work engagement among rural and remote nurses who provide H/P/EOL care. These included: higher perceived mental health (*β* = 0.192, *p* < 0.001), higher satisfaction with their current nursing practice (*β* = 0.156, *p* = 0.019), greater interprofessional collaboration (*β* = 0.135, *p* = 0.019), higher affective organizational commitment (*β* = 0.158, *p* = 0.013), lower continuance organizational commitment (*β* = −0.122, *p* = .021), higher normative organizational commitment (*β* = 0.131, *p* = .029), and lower job demands related to working conditions (*β* = −0.148, *p* = 0.019).

**Table 4:** Predictors of work engagement among rural/remote nurses who provide H/P/EOL care.

Variables	*B*	*SE*	Beta	t	Sig	R^2^ (adjusted)	95% CI
(Constant)	20.062	6.382		3.144	0.002	0.398^a^	
Perceived mental health	2.103	0.626	0.192	3.362	**<0.001**		(0.87,3.33)
Interprofessional collaboration	0.882	0.373	0.135	2.368	**0.019**		(0.15,1.62)
Work community satisfaction	−0.114	0.688	−0.010	−0.165	0.869		(−1.47,1.24)
Community cohesion	0.113	0.087	0.075	1.298	0.196		(−0.06,0.28)
Affective commitment to the organization	0.268	0.107	0.158	2.495	**0.013**		(0.06,0.48)
Continuance commitment to the organization	−0.215	0.092	−0.122	−2.326	**0.021**		(−0.40,-0.03)
Normative commitment to the organization	0.234	0.106	0.131	2.199	**0.029**		(0.02,0.44)
Job satisfaction	1.582	0.673	0.156	2.352	**0.019**		(0.26,2.91)
Perceived stress	−0.213	0.192	−0.066	−1.114	0.267		(−0.59,0.16)
Burnout	−0.490	0.414	−0.071	−1.183	0.238		(−1.31,0.33)
JRIN Supervision, recognition, and feedback	0.204	0.141	0.087	1.444	0.150		(−0.07,0.48)
JRIN Staffing and time	0.026	0.143	0.011	0.180	0.857		(−0.26,0.31)
JRIN Autonomy	−0.312	0.177	−0.110	−1.764	0.079		(−0.66,0.04)
JDIN Preparedness	0.242	0.248	0.052	0.975	0.330		(−0.25,0.73)
JDIN Working conditions	−0.531	0.225	−0.148	−2.363	**0.019**		(−0.97,-0.09)

^a^Dependent variable: Work Engagement

Bold font indicates significance level of *p* ≤ 0.05

Regression n = 255, Cases with missing data on any model variables were excluded

Note: Collinearity diagnostics indicated no problematic multicollinearity; all tolerance values were above  .20 and all VIF values were below 5

### H/P/EOL care in rural and remote settings: Open-ended responses

A total of 184/295 nurses who provide H/P/EOL care (62%) answered the open-ended questions regarding what it means to be a nurse and shared experiences in rural/remote nursing practice.

#### Sense of privilege and humility in providing rural palliative and end of life nursing care.

The open-ended data reflected both a sense of both privilege and humility in providing H/P/EOL care in rural and remote practice settings.

*We provide very good care and most families and clients feel the care they receive here is second to home. We also provide excellent palliative care for clients at the end of their lives – families are encouraged to participate and stay with their loved ones as much as they want to. (Rural RN 211227)*

*The family & loved ones appreciated our efforts…[from] delivering happy screaming babies to a deceased 17 week into my hand…And when I am crying as a deceased community member takes his/her last breaths, I am in the right place… It really doesn’t matter how old or young or where we live.... we are all family and human. (Rural RN 1511782).*

*Working in rural areas as a palliative care nurse and going to patient’s homes has humbled me. I have seen many situations where families live in poverty. I have been exposed to many families and am always touched by their humility and dignity. I never forget that it is a privilege to be part of their journey, that it is a privilege to be allowed in their private and difficult life. (Rural RN 311246)*

*People remember for years what you did for them or their family. What we take for granted, and do as part of our job – people believe we did something outstanding. It is a privilege to share in the new beginnings of life and help people at the end of their life – people we have known for years. (Rural RN 1011153)*

#### Rural specific challenges: Workload, blurred boundaries, lack of education and resources, isolation, and emotional toll.

The barriers and challenges described by rural nurses who provide H/P/EOL care included issues such as workload and staff shortages*: “Significant work overload, need to work fast and always faster…we take care of people, not things, and families that lose people who are important to them, it is becoming dangerous” (Rural LPN/RPN 523124), “There is a daily struggle with lack of support staff…Any day I could be [caring for] a laboring women, followed it with palliative patient, converse with someone who is mentally ill, and take care of a postoperative patient” (Rural RN 1511144);* blurred personal/professional boundaries: *“We deal with death of elderly residents which again is difficult because we know everyone personally” (Rural LPN 813425),*

*I had to care for a man who was just one year younger than me and in addition, I had been his colleague working in another [region] nearby. Earlier, I had his father as a patient in palliative care and I had known and provided support for the patient’s wife and her family and now, the same son, five months later, arrives as a patient in his turn in palliative care with terminal pancreatic cancer. (Rural LPN/RPN 523399)*

*We have recently has several deaths in our community of people in their middle age. While I think we provide good care, it was difficult emotionally. It is very difficult to “leave your work at work”; there is no anonymity. (Rural Registered Psychiatric Nurse 814192)*

and increasing complexity with limited education surrounding pain and symptom management and lack of access to palliative services and resources in general:

*Sometimes they require more complex care (i.e., intrathecal catheters for pain management) and no one in their local community has the proper training to care for them… We need to use resources available to us in creative ways so we can impact as many people as possible. (Rural RN 611356)*

*It is difficult when dealing with palliative clients. There are no services to support these people on weekends or after hours so they call the nurses from the community. It is a challenge to get the proper pain meds in a rural community for the client. We have to jump through hoops to get the proper supplies and meds on time and regularly. (Rural LPN/RPN 713164)*

*Some weeks, no doctor is available for house calls when we have patients who are approaching the end of their lives. It is difficult sometimes because we have a lot of responsibility on our shoulders. We have to see to pain management, respiratory distress, the patients…and we have to deal with the questions and do a lot of training for family members. (Rural RN 521288)*

These barriers are compounded by the generalist nature of rural and remote nursing practice (e.g., care from birth to death), geographical isolation, and vast distances to larger referral centres.

*Being a rural nurse means being able to adjust to an ever changing day. For example, my day could start with a couple of patients waiting for placement, an acute patient waiting for a bed at a higher level of care, a convalescing patient and a palliative patient. In the middle of the day an obstetrics patient can walk in, and/or I have to admit patients from Emergency…The actual nursing work is difficult due to the lack of supervisor support … workload, and at times the delay in accessing resources in other communities. Dealing with transport is extremely time consuming and that takes away from direct patient care. (Rural RN 1011195)*

*They appreciate that we go to see them even if they are far away, if the roads are snowed in or even flooded. We support those who want us to until the end of their lives at home. (Rural RN 521279)*

*We must be a Jack of all Trades Master of None. While the plan is always to stabilize the patient and get them to a larger facility, but with staffing/transport/weather, this is not always possible… Patient care definitely suffers, although the nurses pull together and do the best we can. (Rural LPN/RPN 613518)*

Many respondents detailed intricate stories of knowing their patients/clients as community members, friends, and neighbours, highlighting the additional mental and emotional toll of this work;

*I have worked with many palliative patients and families over the years. I helped a family I am close to with three of their relatives passing. Though it was emotionally exhausting and I felt I couldn’t do it. I managed to make the difficult experience easier for them. I was able to help them understand each stage of palliation, how different medications help with different concerns. These experiences helped me fell like I tried to make this very difficult experience for the family more bearable. (Rural LPN/RPN 913180)*

*[For a] patient near the end of his life… That the family is very upset by the situation...they are not ready to face death. Well, by explaining the situation and listening and by my presence in the face of that, I think it helps certain families. Mentally I am sometimes very tired. (Rural LPN/RPN 523243).*

#### Rewarding and interconnected nature of palliative and end of life care for rural nurses.

Although, the mental and emotional toll of this work was evident, the stories they shared prioritized the rewarding nature of their practice and the importance of the deep connections that they developed with patients and their family members in the role of providing H/P/EOL care in rural and remote settings.

*Being ‘small town’ everyone is somehow connected…The family who comes…who need to say goodbye. The hallway and chapel are filled her friends and family. So my role becomes one of support for family…It is maintaining my composure while watching family members mourn the loss of their loved one. It is also waiting for everyone to leave then phoning the funeral home. It is preparing the body for pick-up. Keeping in mind this is only one patient and there [are] also other patients with needs… Working in a rural setting, you never really know what role you may play. (Rural RN 711313)*

*I can’t count the number of times that I have supported family members during the period when their parents or spouses are dying. Often I let myself feel the suffering caused by their death along with the family members. Often families tell me how much appreciate my being there to share these very intense moments. (Rural RN 521151)*

*We look after our neighbors, our friends, and our family. We dedicate extra unpaid time because of that. We deal with patients at all different stages of their illness from critical to emergent to sending them home to death. We need to be versatile. We deal with family of the women who serves us a meal at the restaurant and ones who we pay for our groceries and those who look after the death of our parents...My friend is the funeral director who picks up my Dad’s body… We are all intertwined…We need to give the family with the death the support they need. (Rural RPN 613198)*

## Discussion

This study is the first to explore, from a national perspective in Canada, the characteristics and predictors of work engagement among a sample of rural and remote nurses who consider hospice, palliative, and/or end of life care a part of their practice. Our results highlight that only a small proportion (approximately 8%) of the full national sample of rural and remote nurses view H/P/EOL care as part of their practice, however, the specific nature of this area of practice was not defined in the national survey questionnaire. It is likely that a greater number of rural and remote nurse respondents provide supportive and comfort care to those facing life-limiting illness, but may not have defined their overall nursing practice as palliative and/or end of life care. There is further concern that the nursing workforce distribution in rural and remote practice has changed since the time of data collection, with the share of nurses working in these settings declining from 11.1% in 2013 to 9.6% in 2022 [6], and potentially fewer nurses who have the knowledge and experience to provide comprehensive H/P/EOL care. Changes also include increasing reliance on internationally educated nurses (IENs) to support staffing in underserved areas, which may contribute to workforce sustainability but also introduces considerations related to navigating rurality (e.g., geography, culture, community integration) and adjustments to a wider scope of practice with limited educational resources [52]. Evidence also suggests that rural and remote nurses in Canada and internationally are less likely to see themselves as palliative care specialists due to the generalist nature of their work, lack of access to palliative care teams, and geographical isolation [5,53,54]. These results are also in line with those of a recent study on barriers to palliative care in a rural US community where a lack of a clear and well-disseminated definition of palliative care and limited access to end of life care education may impede its recognition and understanding among health care professionals [55].

In the present study, even though faced with lower resources related to staffing, and higher demands related to safety (e.g., experiencing/witnessing violence), the mean level of work engagement among 295 rural and remote nurses who provide H/P/EOL care was 40.3 (possible range of 0–54), which was significantly higher than the mean work engagement of 38.4 for the remaining sample. The summary of open-ended data supported these results, whereas, the rural nurses faced barriers/challenges, but also felt a sense of privilege/reward in providing H/P/EOL care within the context of generalist practice and close-knit ties between neighbours, family, and friends. A systematic review and meta-synthesis emphasize positive factors that influence rural and remote nurses’ engagement including collaboration with supportive teams and mentoring, visible sense of belonging, opportunities for professional development, and appreciation of the local rural culture [56].

The present sample of rural nurses who provide H/P/EOL care were significantly younger, more likely to work in community-based settings, and in the smallest communities by population, which may have had a positive impact on their sense of belonging and connection to a rural identity. Thus, there is potential for these factors (e.g., working in smaller, community-based practice settings) to have a protective effect on their level of work engagement even in the face of other challenges [38]. The open-ended responses (n = 184/295) highlighted a potentially deeper and complex emotional landscape where nurses emphasized their work in H/P/EOL care as a privilege and as a source of connection with their community members. The themes also revealed common geographical and emotional challenges in rural/remote practice, including workload pressures, blurred personal-professional boundaries, limited resources, and isolation [18,57]. Although the sense of geographical isolation as complicating their practice was present within the open-ended data, the Job Demands in Nursing: Isolation subscale was not included in the regression model due to low internal consistency reliability within the full subsample. It is imperative that future studies examining predictors of work engagement among rural and remote nurses include standardized measures related to geographical isolation and/or distance to advance referral settings.

Notably, even when describing moral strain and emotional exhaustion in the open-ended findings, participants reaffirmed the rewarding aspects of their work, suggesting that meaning and connection may coexist with and potentially buffer mental distress in rural and remote practice settings. It is interesting that although perceived mental health was the strongest predictor of work engagement in the regression model, the open-ended findings suggest a non-linear and complex relationship where high emotional demands and high work engagement exist together. This contrasts with research that demonstrates that poorer mental health (e.g., emotional exhaustion, psychological distress) is associated with lower engagement among nurses, while positive mental health supports vigor, dedication, and absorption at work [29,58]. This contrast underscores the importance of considering both the psychological well-being and the relational and moral drivers of work engagement when interpreting occupational outcomes in rural and remote practice settings.

Work engagement among health care professionals is recognized globally as crucial for both organizational success and individual well-being, and is directly linked to the quality of patient care [59]. With regard to exploring a combination of individual, workplace/practice, and community variables, the findings of the present study indicate that 7 out of 15 variables (1 individual, 6 workplace/practice, and no community variables) were significant predictors, accounting for 40% of the total variance in work engagement among rural and remote nurses who provide H/P/EOL care. Higher perceived mental health as an individual variable and higher satisfaction with the current nursing practice (i.e., job/work satisfaction) as a workplace/practice variable had higher power in predicting work engagement among this sample. While causation cannot be inferred, the results are consistent with prior research showing that distress and emotional exhaustion undermine nurses’ work engagement [59–61], whereas work satisfaction supports work engagement [60,62]. Although the two above predictors were categorized as an individual and a workplace/practice variable, one cannot ignore the interconnected nature of rural nurses’ mental well-being and job satisfaction in the context of working in the presence of suffering and death/dying in smaller rural settings. Enhanced access to formal and informal mental health supports may promote and assist in sustaining work engagement.

Although the influence of organizational commitment and work engagement on the well-being and job performance of healthcare professionals is well recognized, there are divergent views on whether work engagement is a precursor to organizational commitment, or whether work engagement is an outcome of organizational commitment [63]. Regardless of the direction of the relationships between these concepts, exploration of specific components of organizational commitment (i.e., beyond affective commitment to the organization) are key to gaining a better understanding of work engagement in the context of human resource planning [63]. Affective organizational commitment focuses on nurses’ emotional attachment and identification with their organization, with the strength of this emotional connection having an impact on an employee’s desire to stay [49]. Whereas, normative organizational commitment arises from an employee’s sense of obligation to stay, often linked to a sense of loyalty, and continuance commitment being defined as a “have to versus want to” commitment, driven by the perceived costs of leaving an organization as being too high or due to a lack of alternatives (e.g., difficulty finding a comparable job) [49]. The results of the present study showed that higher affective commitment to the organization, lower continuance commitment, and higher normative commitment to the organization were significant predictors of work engagement among rural and remote nurses who provide H/P/EOL care. Considering the emotionally demanding context of providing palliative and end of life care in rural settings, the importance of affective commitment to the organization as a predictor of higher work engagement is especially relevant in the present study. Higher normative commitment as a predictor of work engagement highlights the importance of having a sense of loyalty or feeling obliged to stay within the organization for the present sample. However, it is noteworthy that lower continuance commitment was also a significant predictor of work engagement, suggesting that the present sample may have been less driven by the perceived costs of leaving, and they did not necessarily have fewer options to seek employment in other areas of practice.

Although the rural and remote nurses in the present sample were more likely to work in the smallest communities, having greater interprofessional collaboration was a significant predictor of work engagement among those providing H/P/EOL care. In the last decade, the growing use of virtual care and increased access to communication technologies in rural and remote settings may facilitate interprofessional collaboration and reduce professional isolation, particularly in settings with limited specialty resources [64,65]. In relation to evidence-based models of rural palliative care, Marshall and colleagues highlighted the crucial element of having prepared, informed, and motivated health care professionals in a well-coordinated team approach lead by those with specialty expertise in palliative care as key to improved outcomes in rural practice settings [16]. The reality is that most rural and remote settings are limited in access to local specialty palliative care resources, however, research indicates that these communities can build capacity by capitalizing on close-knit, committed palliative practitioners and by promoting remote education and support to increase expertise within health care teams [4].

The absence of community-level predictors in this model of work engagement was unexpected given their theoretical significance [56,66,67]. However, in the open-ended responses nurses described their work as deeply meaningful and rooted in their relational and community connections. Although not a community-level predictor, lower job demands related to working conditions emerged as a significant predictor of work engagement and may indirectly reflect the benefits of community-based practice environments. This finding is particularly relevant in the context of ongoing challenges with recruitment and retention of rural and remote nurses in Canada. A historical narrative review highlights that these challenges have persisted and worsened since the early 20^th^ century, with workload and burnout remaining as central issues [68]. High job demands and poor working conditions not only undermine engagement but also contribute to difficulties in sustaining the workforce. Conversely, evidence from a structural equation modeling study exploring the relationships between palliative nurses’ mental health, physical health, work engagement, and job crafting (e.g., proactive altering of job resources and demands) indicates that those nurses who are able to modify their job demands and resources experience higher work engagement, better physical health outcomes, and lower levels of mental health distress [69]. Although identifying which specific job demands related to working conditions are modifiable is beyond the scope of this study, these findings underscore the importance of addressing working conditions as part of broader recruitment and retention strategies, especially in light of the current rural and remote workforce distribution. Further research is necessary to determine what organizational and policy-level actions are more likely to lower rural and remote nurses’ job demands, improve working conditions, and potentially lead to higher engagement in palliative nursing practice in these settings.

## Limitations

There are a few limitations that should be considered regarding this study. First, due to the cross-sectional nature of the data collection and analyses based on correlational relationships between variables, causal relationships between the predictor variables and the outcome variable of work engagement cannot be established. Second, the data for this study were collected prior to the start of the COVID-19 global pandemic which had a significant impact on health care professionals, populations, and communities in both rural and urban settings. Nurses who were practicing throughout the pandemic were often under-resourced, faced high demands, and were thrust into having to provide palliative and end of life care with limited expertise and support. As well, the nursing workforce distribution in rural and remote practice has worsened since the time of data collection. Therefore, the findings of this study should be generalized with caution regarding post-pandemic rural practice settings and recognition of the persistent challenges in recruiting and retaining rural and remote nurses in Canada. Finally, the full national study covered a broad spectrum of rural and remote nursing practice, with limited focus on defining the nature of hospice, palliative, and end of life care. The operationalization of our subsample was limited by use of a single self-report area of current practice item, with hospice/end of life/palliative care being one of seven areas and the sample being asked to ‘mark all the apply.’ This may have limited the sample size of rural and remote nurses who considered hospice, palliative, and end of life care as part of their current practice. It is likely that a larger proportion of rural and remote nurses provide palliative and supportive care (e.g., pain and symptom management, emotional support, education regarding death/dying) to patients with terminal or life-limiting illness and their family members, especially in a post-pandemic context.

## Conclusions

This is the first Canadian national profile of rural and remote nurses who provide hospice and palliative care and highlights key areas related to their professional quality of life (e.g., work engagement, job satisfaction, organizational commitment, job/practice resources and demands). Although there was a delay between the research data collection and dissemination of these results, there is potential for the quantitative results and open-ended findings of this study to still hold relevance considering the lack of high quality published research focusing on this rarely studied population. In particular, this study may provide the foundation for the replication of this research in rural and remote nurses who provide H/P/EOL care in other settings or countries. The results of this analysis may also provide guiding information that could assist in informing practice and policy development for effective health human resource planning and recruitment/retention in hospice, palliative and/or end of life care across rural and remote settings. Although the data were collected prior to the start of the COVID-19 global pandemic, the results highlight the lack of research in this area and the need for further exploration and studies designed with a specific focus on H/P/EOL nursing care in rural and remote practice settings.
